# Reconfigurable radio-frequency arbitrary waveforms synthesized in a silicon photonic chip

**DOI:** 10.1038/ncomms6957

**Published:** 2015-01-12

**Authors:** Jian Wang, Hao Shen, Li Fan, Rui Wu, Ben Niu, Leo T. Varghese, Yi Xuan, Daniel E. Leaird, Xi Wang, Fuwan Gan, Andrew M. Weiner, Minghao Qi

**Affiliations:** 1 School of Electrical and Computer Engineering and Birck Nanotechnology Centre, Purdue University, 1205W. State Street, West Lafayette, Indiana 47907, USA; 2 State Key Laboratory of Functional Materials for Informatics, Shanghai Institute of Microsystem and Information Technology, Chinese Academy of Sciences, 865 Changning Road, Shanghai 200050, China

## Abstract

Photonic methods of radio-frequency waveform generation and processing can provide performance advantages and flexibility over electronic methods due to the ultrawide bandwidth offered by the optical carriers. However, bulk optics implementations suffer from the lack of integration and slow reconfiguration speed. Here we propose an architecture of integrated photonic radio-frequency generation and processing and implement it on a silicon chip fabricated in a semiconductor manufacturing foundry. Our device can generate programmable radio-frequency bursts or continuous waveforms with only the light source, electrical drives/controls and detectors being off-chip. It modulates an individual pulse in a radio-frequency burst within 4 ns, achieving a reconfiguration speed three orders of magnitude faster than thermal tuning. The on-chip optical delay elements offer an integrated approach to accurately manipulating individual radio-frequency waveform features without constraints set by the speed and timing jitter of electronics, and should find applications ranging from high-speed wireless to defence electronics.

## Abstract (web summary)

Performing radio-frequency arbitrary waveform generation in the optical domain offers advantages over electronic-based methods but suffers from lack of integration and slow speed. Here, Wang *et al*. propose a fast-reconfigurable, radio-frequency arbitrary waveform generator fully integrated in a silicon chip.

## Graphical abstract

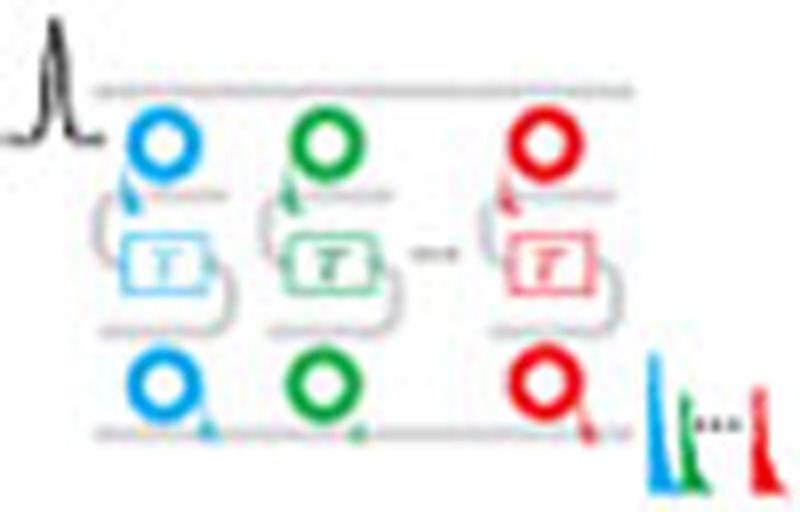

Arbitrary waveform generation (AWG) is critical to many radio-frequency (RF) and time-domain tests, measurements and applications, including ultrawide-bandwidth and multiple-access wireless communications, electronic counter-measures and radar systems. Commercial electronic methods of AWG, while continually improving, are limited by the speed and linearity of digital-to-analogue converters. Photonic methods of generating and processing RF signals have offered high bandwidth, intrapulse modulation and low phase noise that are difficult to obtain by electronic means^1^,^2^,^3^,^4^,^5^,^6^. They typically involve the generation of delayed and scaled replicas of RF signals or impulses in the optical domain with subsequent recombination and scaling to achieve the desired RF output (Fig. 1a). Conventionally, multiple optical carriers at different wavelengths go through a pulse shaper^7^ to acquire the desired amplitude and/or phase changes.

With commercial electronic AWG improving in speed and timing, photonic AWG must increase the reconfiguration speed^8^, duty cycle and system stability, as well as reduce the cost, size, weight and power consumption. One of the most promising solutions is the miniaturization and integration of photonic devices. To that end, photonic beamformers for phased array receive antennas^9^,^10^, fractional Hilbert transformers^11^,^12^, and so on, have been successfully demonstrated in indium phosphide (InP), silica on silicon, silicon nitride or hybrid silicon platforms. In addition to optical/RF waveform generation and signal processing^13^,^14^,^15^,^16^,^17^, integrated devices may find applications in RF photonics^18^ and microwave photonic filters^19^,^20^,^21^,^22^,^23^,^24^,^25^,^26^. Each material platform has its own advantages and drawbacks, with InP currently leading some performance metrics. However, silicon photonics offers the lowest cost and highest production volume thanks to its compatibility with robust complementary metal–oxide–semiconductor (CMOS) manufacturing.

We have first demonstrated a silicon on-chip pulse shaper—a multi-channel reconfigurable microring filter bank—and used it to generate eight replicas of RF impulses at eight tuneable optical frequencies from an ultra-fast optical pulse and to scale them to specified amplitudes before recombination^27^. However, to achieve appropriate delays between the eight replicas, the recombined signals need to go through a dispersive device, such as a long spool of optical fibre (5.5 km long^27^) or a linearly chirped fibre Bragg grating^5^,^28^. Such a process is called frequency-to-time mapping^1^,^2^, which provides accurate and continuous adjustment of delays, a feature difficult to achieve by electronic means if delay differences between adjacent carrier wavelengths are as short as a few tens of picoseconds. Unfortunately, dispersive devices that can provide strong dispersion over a wide optical bandwidth of tens of nanometres are not amenable to integration in a silicon platform. For example, to achieve the same amount of dispersion as in a 5.5-km-long fibre, a 21-m-long silicon waveguide with a dispersion coefficient of 4,400 ps km^−1^ nm^−1^ is required^29^. The resulting high propagation loss and large footprint (cm^2^) make this option impractical. Chirped Bragg gratings are capable of providing larger dispersion coefficients but are further constrained by the limited delay-bandwidth products in on-chip implementations^30^,^31^,^32^,^33^. Photonic crystals show good performance in delaying optical signals but still impose significant challenges in fabrication^20^,^34^,^35^.

Here we introduce the architecture of RF AWG based on direct time-domain synthesis in a silicon platform that eliminates the requirement of a highly dispersive and physically long medium. Replica pulses associated with different carrier wavelengths are separated in the time domain via multiple on-chip tuneable optical delay lines. Compared with previous frequency-domain approaches, the time delays are no longer tied with carrier wavelengths and the delay tuneability can improve the flexibility in frequency and phase control of RF waveforms over previous time-domain approaches^5^,^15^,^16^. The quality of the generated RF bursts or continuous RF waveforms is comparable to that of the waveforms demonstrated with the frequency-domain scheme based on frequency-to-time mapping^27^. Moreover, certain features of the generated RF waveforms are dynamically reconfigured at nearly gigahertz speed using the fast free-carrier depletion effect in silicon, demonstrating an advantage over photonic RF generation and signal processing reported in the SiO_2_ or Silicon Nitride platform^15^,^19^,^36^.

## Results

### Time-domain RF synthesis using Si optical delay lines

**Figure 1:**
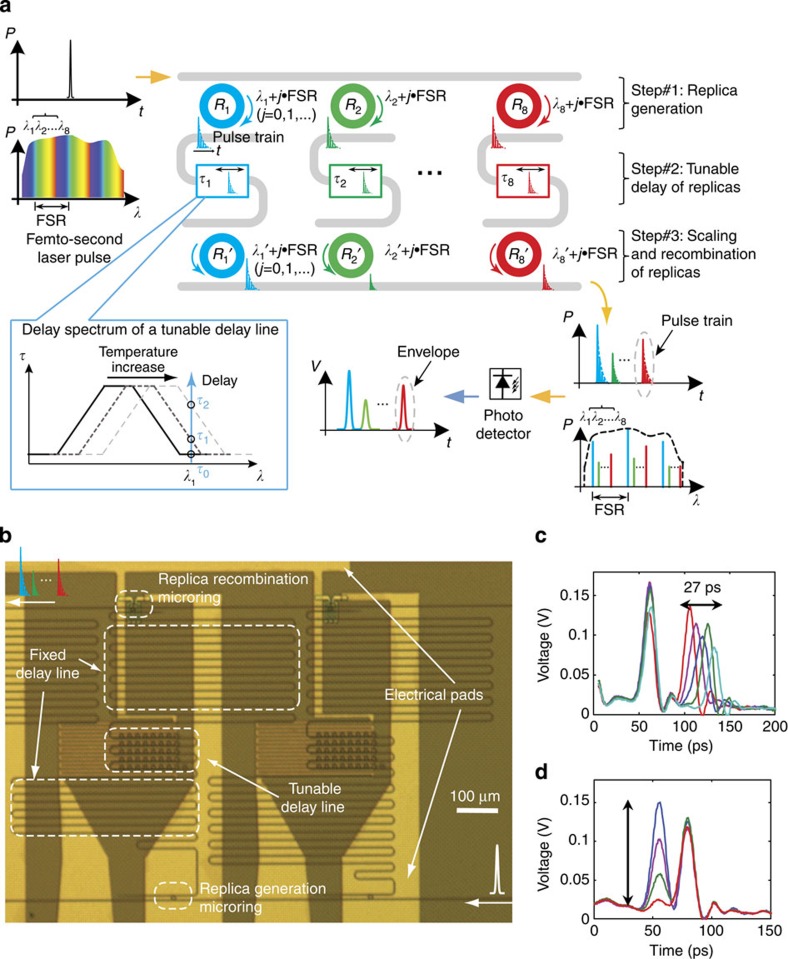
Time-domain synthesis of RF waveforms using a Si photonic chip. (**a**) Schematic of the time-domain synthesis method where a femtosecond laser pulse is replicated, delayed and recombined through an eight-channel Si pulse shaper. In the spectrum representation of the input pulse, wavelength components that correspond to different resonances of a replica generation microring are labelled using the same colour, and are downloaded by that same microring from the input waveguide to form a channel replica pulse. The eight pulse replicas experience various tuneable delay and are separated in the time domain. The amplitude of each pulse replica can be adjusted during the recombination process by mismatching the resonance of the recombination microring against that of the replica generation ring. Finally, the properly delayed and scaled replicas are recombined at the common output port of the shaper to form the desired optical waveform. An off-chip photodetector detects the envelope of the output optical waveform, producing the synthesized RF waveform. The inset schematically shows the mechanism of the thermally tuneable optical delay lines. FSR, free spectral range of a microring. (**b**) An optical image of a fabricated pulse shaper. (**c**,**d**) Experimental demonstrations of ~27 ps delay tuning and complete amplitude control of a channel replica pulse without appreciably affecting the pulse from an adjacent channel.

Figure 1a depicts the principle of time-domain RF waveform synthesis, where a femtosecond laser pulse is tailored by a multi-channel (eight in Fig. 1a) silicon pulse shaper in both the frequency and time domains and is converted to a RF waveform using a photodetector. First, an input ultra-fast pulse (~130 fs, ~80 nm bandwidth) is sampled by eight microring add-drop filters of different radii *R*_*i*_ (*λ*_*i*_ denotes the associated resonance wavelength, *i*=1, 2,..., 8) that download and produce eight replica pulses. Since the wide bandwidth of the input pulse covers six free spectral ranges of the 5-μm radius silicon microrings (free spectral ranges=~16 nm), each replica generation microring downloads up to six small slices of the input spectrum. In the time domain, each pulse replica actually consists of a series of densely packed yet separated pulses of exponentially decaying amplitude (See Supplementary Fig. 1). However, only the envelopes of the pulse replicas can be detected during optical/electrical conversion and are of interest to RF AWG applications. The width of each pulse replica’s envelope is inversely proportional to the optical round-trip time in the ring and the ring’s power coupling coefficients to bus waveguides (See Supplementary Fig. 1). To avoid excessive broadening of the pulse replicas’ envelopes as well as to reduce the insertion loss of the add-drop filters, the microrings are designed to over-couple to the bus waveguides (with a power coupling coefficient of *κ*^2^≈0.1). The frequency spacing between channels in the filter bank can be arbitrary and non-uniform as long as they are not too small to cause interference between channels. In our case, an approximately uniform channel spacing of 1 nm is chosen (with a designed radius step increment of 8 nm) but no effort is put in to accurately achieve it (See Methods and Supplementary Fig. 2). This reduces the fabrication difficulty and thermal tuning power as no tuning of the replica generation ring’s resonance is required.

The key component of photonic AWG is the on-chip tuneable delay element. In our design, each optical delay line consists of a fixed part and a tuneable part (Fig. 1b), and the total time delay in the *i*-th channel of the shaper is given by *τ*_*i*_=(*i*−1) × 25 ps+*τ*_tuneable, *i*_ (*i*=1, 2,…, 8). The fixed part is a long waveguide with a delay value at multiples of 25 ps (group index *n*_g_≈4.2 for the transverse-magnetic (TM) mode), and each channel has 25 ps more delay than the previous one. The tuneable part consists of 41 cascaded microring all-pass filters, the radii of which are designed to monotonically increase by a 1-nm step. The median radius of the microrings in the delay line is designed to be ~32 nm smaller than that of the replica generation ring in the same channel. Consequently the tuneable delay is at its minimum value when no external thermal tuning is applied (See the inset of Fig. 1a and Supplementary Fig. 3). All the microrings in the tuneable delay line are designed in the overcoupling regime (with a power coupling coefficient of *κ*^2^≈0.6) to mitigate dispersion-induced pulse distortion as well as to reduce the insertion loss^37^,^38^. The delay spectrum of the tuneable delay part is schematically shown in the inset of Fig. 1a and tuning of the delay at *λ*_1_ can be achieved by shifting the entire spectrum. A serpentine micro-heater covering the tuneable delay line area (Fig. 1b) is employed to thermally tune the resonances of all the 41 rings, thus shifting the spectrum. The simulated delay spectrum of the microring array shows a delay range of up to *τ*_tuneable,*i*_≈37 ps at an additional insertion loss of 3 dB (See Supplementary Fig. 3). In the experiment, a tuneable delay range of up to *τ*_tuneable,*i*_=27 ps, greater than the fixed delay step of 25 ps between two adjacent channels, was observed (Fig. 1c) with an additional insertion loss <3 dB, which is a consequence of the time delay, caused by additional propagation loss when light travels longer within the ring array. Due to the limited control one can achieve in materials and fabrication, deviations in the microrings’ resonances from their designed values are inevitable. However, according to our simulation the characteristics of the tuneable delay line are not appreciably affected by such deviations (See Supplementary Fig. 3). This fabrication tolerance is an important feature of our design and may allow future high-volume manufacturing.

The amplitude of each replica pulse can be controlled by mismatching the resonance wavelength *λ*_*i*_ of the replica recombination ring from that of the replica generation ring in the same channel through the thermo-optic effect. The perimeter of the replica recombination ring is designed to be slightly smaller than that of the replica generation ring (See Methods and Supplementary Fig. 2), and micro-heaters are placed on top of each recombination ring only. Figure 1d shows almost 100% amplitude control of a replica pulse without significantly affecting the reference pulse in an adjacent channel.

### Synthesis of RF bursts

**Figure 2:**
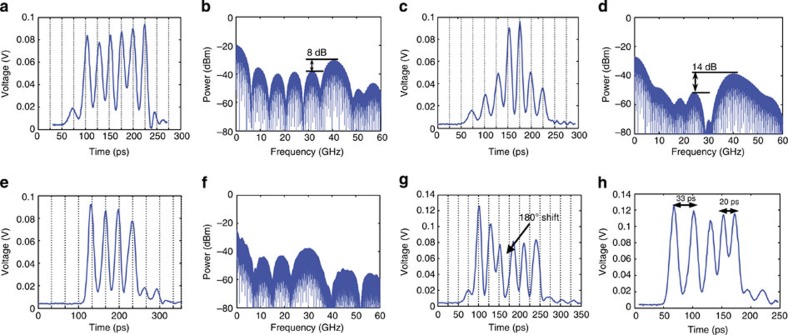
Demonstrated RF bursts and their spectra. (**a**,**b**) Experimentally demonstrated a 40-GHz RF burst and its spectrum calculated via Fourier transform, where the mainlobe–sidelobe suppression ratio (MSSR) is ~8 dB. (**c**,**d**) An apodized 40-GHz RF burst and its calculated spectrum, where the MSSR increases to ~14 dB. (**e**,**f**) A 30-GHz RF burst and its calculated spectrum. (**g**) *π* phase shift in a 40-GHz RF burst. (**h**) An up-chirping signal with frequencies ranging from 30 to 50 GHz.

With the capabilities of independent amplitude and delay control in the shaper, RF waveforms with sophisticated amplitude, frequency and phase modulations were created (Fig. 2). The first example of a 40-GHz RF burst (Fig. 2a) was created with the preset channel delay spacing of 25 ps by the fixed delay lines and with a slight tuneable delay applied for minor adjustment. By apodizing the envelope of the 40-GHz burst to a smooth shape (Fig. 2c), the mainlobe–sidelobe suppression ratio in the corresponding RF spectrum was improved from 8 dB (Fig. 2b) to 14 dB (Fig. 2d). The next three examples are a 30-GHz RF burst, a 40-GHz RF burst with an abrupt *π* phase shift and a frequency-chirped signal (Fig. 2e,g,h, respectively). The chirped signal shows a frequency modulation from 30 to 50 GHz, containing very broadband frequency components, which can be useful in chirped radar systems. The time-domain approach produces ultrabroadband RF arbitrary waveforms with performance comparable to our previous demonstration where we used a Si photonic pulse shaper chip plus external frequency-to-time mapping^27^, but without the need for an optical fibre of multi-kilometre length.

### Synthesis of continuous RF waveforms

**Figure 3:**
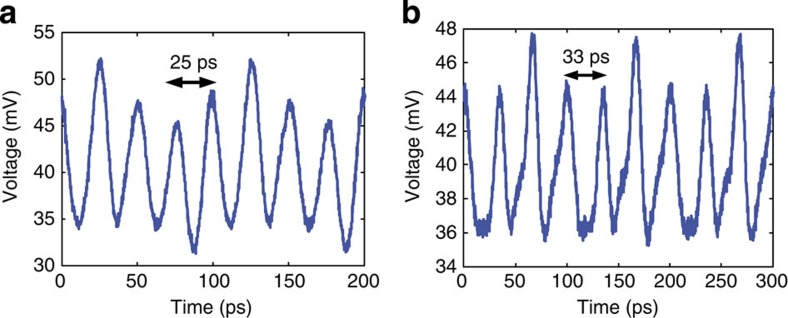
Single-tone continuous RF waveforms. (**a**) A 40-GHz continuous RF waveform and (**b**) a 30-GHz continuous RF waveform are generated using a 10-GHz optical frequency comb source.

The RF waveforms demonstrated thus far have been in the form of repetitive RF bursts, each synchronized with a particular pulse of the low repetition rate input femtosecond pulse laser. Consequently the duty cycle is low. These RF waveforms are inefficient for communications and information processing where high data throughput is paramount. To obtain RF waveforms with high duty cycles, one can either use a laser source with a higher repetition rate that is close to the inverse of the temporal width of the bursts or introduce more features in one RF burst. Here we adopted the first scheme and chose a 10-GHz optical frequency comb as a new light source to generate continuous RF waveforms^39^. The optical frequency comb is generated from a continuous-wave (CW) laser source based on periodic electro-optic modulation^40^. The carrier wavelength of 1,542 nm of the comb coincides with the wavelength range of the pulse shaper, and the comb spans a 6-nm wavelength range covering >4 channels of the shaper. Figure 3a shows an example of a 40-GHz continuous RF waveform where four channels were selected with the channel delay spacing preset at 25 ps. A 30-GHz continuous waveform was created when three channels were selected with the channel delay spacing adjusted to 33 ps through a tuneable delay (Fig. 3b). Therefore, single-tone continuous RF waveforms can be generated using a high repetition rate light source and the photonic chip with a properly preset channel delay.

### Rapidly reconfigurable waveform enabled by a Si modulator

Truly arbitrary RF waveforms allow modulation of features in a pulse-by-pulse fashion, which can take the full advantage of the wide bandwidth of RF waveforms^5^,^14^. All the above-mentioned reconfiguration of the waveform generator is achieved through the thermo-optic effect in silicon, which has a response time around tens of microseconds^41^. While this is already faster than bulk pulse shapers employing liquid crystal modulators, in practice the fast reconfiguration time close to the inverse of the waveform repetition rate is desirable for many applications such as wireless data communications. An advantage of silicon over liquid crystals or other materials in photonic integrated circuits, such as SiO_2_, is that it offers a strong free-carrier effect with a sub-nanosecond response time^42^. Here we employ a free-carrier depletion effect based Mach–Zehnder interferometer intensity modulator to demonstrate rapid amplitude control of an individual pulse^43^,^44^,^45^,^46^.

**Figure 4:**
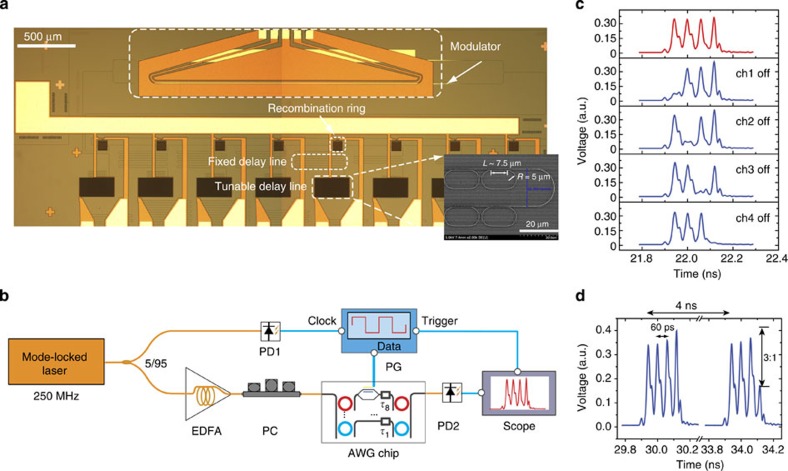
Rapid waveform reconfiguration using a silicon modulator. (**a**) An optical image of a rapidly reconfigurable AWG chip fabricated in a CMOS manufacturing foundry. The AWG chip consists of an eight-channel silicon pulse shaper and a silicon free-carrier depletion effect based intensity modulator embedded in the 8th channel of the shaper. The replica generation rings are not shown in the optical image. The inset is a scanning electron microscope picture of a tuneable delay line. (**b**) Experimental set-up. PC, polarization controller; PD, photodetector; PG, pattern generator. (**c**) Amplitude control of individual pulses through resonance mismatching between the replica generation and recombination rings. ch1, channel 1. (**d**) Two consecutive RF bursts show rapid amplitude modulation of the last pulse in the waveforms through a synchronously driven silicon intensity modulator.

Figure 4a is an optical image of the silicon chip for rapidly reconfigurable RF AWG, which was fabricated in a CMOS foundry using 0.13-μm optical lithography. The silicon chip consists of an eight-channel pulse shaper and an intensity modulator embedded in the 8th channel of the shaper. For proof of concept, only one modulator is incorporated. Since the modulator is designed for transverse-electric (TE)-polarized light, all the microrings in the shaper need to work in the overcoupling regime for TE light. For a 5-μm radius microring, this would require a coupling gap of <100 nm, which is beyond the capability of the optical lithography that we have access to. To overcome this challenge, we designed a 5-μm radius racetrack microresonator with a long coupling region (~7.5 μm) to the bus waveguides so that a coupling gap of 200 nm can still achieve overcoupling (Fig. 4a). We retrieved the *Q*-factor, free spectral range and ring-waveguide power coupling coefficient from measured transmission spectra, compared them with the simulated values for both TE and TM polarization modes using a finite-difference time-domain method^47^, and confirmed that the shaper worked in TE polarization (See Supplementary Table 1). With a 250 MHz optical frequency comb source, we first created 16-GHz RF bursts by selecting four channels in the shaper without fast modulation. The amplitude of each pulse in the created waveform can be controlled completely and individually without appreciably affecting other pulses (Fig. 4c). Next we modulated the intensity of the pulse from the last channel. To synchronize the modulation with the created RF waveform, we converted 5% of the input pulse power to an electrical signal that served as the clock signal of the pattern generator, as shown in the experimental set-up in Fig. 4b. Two consecutive RF waveforms separated by ~4 ns clearly show the fast reconfiguration of the last channel pulse (Fig. 4d). The modulation depth of ~3:1 is consistent with that measured with a CW laser (See Supplementary Fig. 4).

The update speed is currently limited by the repetition rate of the comb laser source as well as the design of the modulator embedded in the shaper (See Supplementary Fig. 4). A 44-Gbps modulation has been demonstrated in the modulators fabricated using the same process^48^, so in principle the reconfiguration time of RF waveforms can reach the deep sub-nanosecond range and continuous RF waveforms with intensity modulation at any pulse can be achieved. For single-tone continuous RF waveforms, our scheme allows the modulators to be run at a speed reduced by a factor equal to the number of peaks per burst, with significantly relaxed timing requirements. Rapid tuning of the delay is also necessary. This could be achieved by directly modulating all microring all-pass filters in a tuneable delay line that share a common RF driving signal. Recently, a cascaded-ring modulator has been demonstrated with a 0.2 nm bandwidth, allowing small distortion to the RF signals^49^.

## Discussion

A dispersive device, such as a spool of optical fibre or a fibre Bragg grating, has been one of the central elements that allow photonics to process microwave signals. Therefore it is almost ubiquitous in microwave photonic processing systems. However, direct integration of this element onto a silicon chip will lead to prohibitively long waveguides and/or intolerably high insertion loss. Our approach solves this problem by controlling the optical delay of each group of wavelength components on the chip in the time domain. Continuous tuning of delays between channels is possible as long as the delays are not less than the fixed delays, which in our case is 25 ps, (see Fig. 2e and Supplementary Fig. 5). While a sparse number of delays <25 ps might be accommodated, for example, in Fig. 2h, uniform delays substantially shorter than 25 ps, for example, 20 ps, cannot be achieved in our current set-up. Nevertheless, this can be straightforwardly solved by reducing the digitization step or the fixed delay between channels. To achieve the same total delay, the number of channels needs to increase, and fortunately this is much easier to achieve in the integrated form than in bulk optics. For example, an impressive arrayed waveguide grating accommodating one hundred 10 GHz frequency channels has been reported in an InP platform recently^14^. Therefore our approach can be viewed as functionally equivalent to frequency-to-time mapping, and we have demonstrated RF AWG with performance in amplitude, frequency and phase control comparable to those achieved in our previous Si photonic shaper chip employing a long fibre for frequency-to-time mapping^27^. In addition, our approach has the advantage that a channel wavelength is no longer tied to a specific delay value, which offers additional flexibility and ease of fabrication.

The integration of fast silicon modulators with pulse shapers on the same chip allows agility, or speed of reconfiguration, unachievable in previous demonstrations using either bulk optical pulse shapers or photonic integrated circuits based on silica or SiN^15^,^19^. Such rapidly reconfigurable RF waveforms generated through integrated modulators enable pulse-by-pulse variable arbitrary RF signal generation, a step towards fully dynamic AWG that is highly desirable for radar applications^14^. With the availability of Ge photodetectors^50^, complete on-chip RF generation is possible. We note that significant progress has been made in InP-based photonic integrated circuits for optical AWG, with a potential for high-speed and coherent reconfiguration capability^13^,^14^. However, tuneable optical delays were not implemented, and challenges in bringing high-speed electrical control signals onto the chip have so far restricted the rapid reconfiguration potential. The ultra-compact tuneable optical delays and pulse shaper, as well as the low-cost and high-yield manufacturing demonstrated here, together with the prospect of seamless integration with on-chip control electronics, make Si-based RF photonic solution an attractive alternative to existing integrated RF photonic platforms. Our approach should also be applicable to a variety of microwave photonic applications beyond the AWG application discussed here, including RF photonic filters^51^,^52^,^53^, photonic beamformers for phased array receive antennas^9^,^10^ and precompensation of dispersive microwave antenna links^54^.

## Methods

### Design and fabrication of the preliminary chip

The waveguides in the preliminary pulse shaper (Fig. 1) fabricated at Purdue have a cross-section of 500 nm × 250 nm (width × height). The radii of replica generation (recombination) microrings start at 5.01 μm (5 μm), with a step increment of 10 nm (8 nm) to separate its resonance from that in the previous channel by ~1.25 nm (~1 nm). The add-drop rings couple to both bus waveguides at the same coupling gap of 200 nm to satisfy the overcoupling condition (with a power coupling coefficient of *κ*^2^≈0.1). Each delay line consists of a fixed part and a tuneable part. The fixed part is a long waveguide with a length multiples of 1.74 mm and each one is 1.74 mm longer than the one in the previous channel. The tuneable delay line contains 41 all-pass microrings that side couple to the common bus waveguide with the same targeted coupling gap of 100 nm (with a power coupling coefficient of *κ*^2^≈0.6). The chip was fabricated on a silicon-on-insulator wafer with high resolution electron-beam lithography. We wrote the microring resonators with the smallest beam step size (1 nm) to achieve a high resolution and wrote the straight waveguides and fixed delay lines with a larger beam step size (12 nm) to save electron-beam lithography writing time. After the silicon waveguide and resonators were defined, a 2-μm-thick silicon dioxide layer was deposited over the device as a buffer layer, using plasma enhanced chemical vapour deposition. To smooth the surface of the oxide layer, we spin-coated a layer of hydrogen silsesquioxane and annealed it to form a planarized film with a thickness of 500 nm. The electrical heaters were then defined by optical lithography. The compact heaters on top of the microrings were made of 150-nm-thick titanium, since the large resistivity of titanium can achieve a high resistance with 3-μm-wide metallic wires, which is critical for fine tuning of microrings’ resonance wavelengths. The serpentine long heaters on top of the tuneable delay lines were made of 300-nm-thick aluminium together with the contact leads.

### Characterization of the preliminary chip

We first measured the transmission spectra at the common through port of the replica generation rings and that of the replica recombination rings using a tuneable CW laser before thermally matching the resonances (See Supplementary Fig. 2a). The averaged channel spacing of the two groups of rings is ~1.4 nm and ~1.1 nm, respectively, and the resonance wavelength of each replica generation ring is shorter than that of the corresponding replica generation ring throughout all the channels as designed. The fibre-to-fibre coupling loss was measured to be ~22 dB, which is mainly attributed to waveguide facet coupling loss. After thermal matching of resonances, the transmission spectra were taken at both the through port and drop port (See Supplementary Fig. 2b,c). The insertion loss in each channel was determined to range from ~4 to ~6 dB after waveguide facet coupling loss was subtracted. The variation in insertion loss is attributed to the length differences between fixed delay lines. For the tuneable delay line, we applied ~100 mW electrical power to the micro-heater on top of the microring array to achieve a delay of ~27 ps.

### Frequency comb for continuous RF waveform generation

In the experiment of continuous RF waveform generation, the original optical frequency comb generated based on periodic electro-optic modulation spans over a wavelength range of 2.4 nm, which is not sufficient for the RF AWG application. We broadened the comb bandwidth to 6 nm via non-linear propagation in a length of dispersion decreasing fibre, which can cover >4 channels of the shaper^55^. Before the dispersion decreasing fibre, a low-noise erbium-doped fibre amplifier (EDFA) and a high-gain EDFA were used sequentially to boost the comb signal to a power level sufficient for the adiabatic pulse compression.

### Fabrication and measurement of the foundry chip

The device was fabricated in a commercial CMOS foundry using 0.13-μm technology on 8″ silicon-on-insulator wafers with a 220-nm-thick top silicon layer and a layer of 2-μm-thick buried oxide (Fig. 4). The waveguides are 500 nm in width. The racetrack resonator has a bending portion of ~5 μm in radius and a straight portion of ~7.5 μm in length that side couples to the bus waveguide at a coupling gap of 200 nm. We first characterized the foundry chip with a tuneable CW laser and found that the drop-port transmission in the 8th channel of the shaper was 10 dB lower than all the other channels due to the insertion loss of the modulator. To show the fast modulation of the replica pulse in the last channel of RF waveform more clearly, we compensated for this loss by pre-attenuating all frequency components outside the 8th channel in the input pulses by 10 dB using a commercial pulse shaper throughout a wavelength range from 1,528 to 1,568 nm.

The experimental set-up for rapidly reconfigurable AWG is shown in Fig. 4b. The 250 MHz optical pulses were first compressed using a length of ~12.5-m-long dispersion compensation fibre. Five percent of the input optical pulse power was converted to an electrical signal that served as the clock signal of the pattern generator, which drove the intensity modulator working synchronously with the generated RF waveform. An RF amplifier (10–1,500 MHz) was used to boost the electrical signal after the photodetector PD1, which was followed by an RF low-pass filter (DC-400 MHz) to filter out high order harmonics. A post-device EDFA was used to boost the optical output from the chip and a 12-nm 3-dB bandwidth optical band-pass filter was used afterwards to remove the strong amplified spontaneous emission around the 1,530 nm wavelength range from the EDFA.

## Author contributions

J.W. and H.S. designed the devices. L.F. and L.T.V. fabricated the preliminary devices. J.W. and H.S. measured the devices. F.G. designed the on-chip modulator. Y.X. and B.N. helped in the fabrication of preliminary devices. R.W. and D.E.L helped in the measurements. M.Q. and A.M.W. conceived the idea and supervised the investigation. J.W., M.Q., A.M.W. and H.S. wrote the manuscript. All authors discussed the results and commented on the manuscript.

## Additional information

**How to cite this article**: Wang, J. *et al*. Reconfigurable radio-frequency arbitrary waveforms synthesized in a silicon photonic chip. *Nat. Commun.* 6:5957 doi: 10.1038/ncomms6957 (2015).

## Supplementary Material

Supplementary Information Supplementary Figures 1-5, Supplementary Tables 1 and Supplementary References.
