# Supplementary material for: Reconfigurable radio-frequency arbitrary waveforms synthesized in a silicon photonic chip
Source: Nat Commun. 2015 Jan 12;6:5957. doi: 10.1038/ncomms6957 (PMC4354206; doi:10.1038/ncomms6957)
Supplement: Supplementary Information — Supplementary Figures 1-5, Supplementary Tables 1 and Supplementary References. [file ncomms6957-s1.pdf]

Supplementary Figure 1

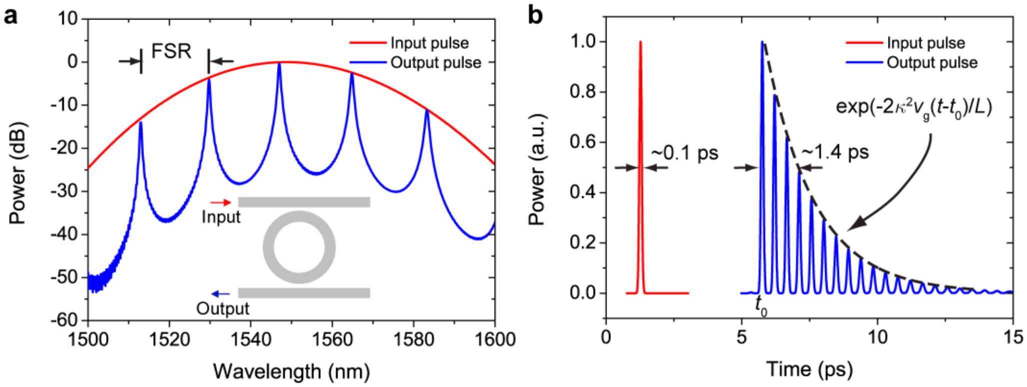

Supplementary Figure 1. Simulation of the broadening of an ultrafast pulse when it transmits through a high-*Q* micro-resonator. We simulate a ~100 fs pulse transmitting through a 5μm-radius, over-coupled silicon microring add-drop filter using a 3D finite-difference time-domain (FDTD) method based numerical solver MEEP[1]. The microring is the same design as that of a replica generation (recombination) ring in the shaper shown in Fig. 1a in the main text, where the ring-waveguide power coupling coefficient is $\kappa^2 \approx 0.1$. (**a**) The optical spectra of the input (in red) and output (in blue) pulses. The input pulse has a center wavelength of ~1550 nm, and its ultrawide spectrum covers multiple free spectral ranges (FSRs) of the microring. (**b**) The temporal profiles of the input pulse (in red) and output pulse train (in blue). The peak value of each pulse in the output pulse train decays exponentially with a time constant of Q/ω_0, where ω_0 is the resonance frequency close to 1550 nm and $Q \approx 2600$ is the corresponding quality factor. In the RF AWG experiment, the photo-detector responds only to the envelope of the output optical pulse train, whose 3dB temporal width is given by $\ln 2 \times Q/\omega_0$. The quality factor Q of the over-coupled microring is related to the power coupling coefficient κ^2 through $\omega_0/Q \approx 2\kappa^2 v_g/L$, where v_g is the group velocity of light in the waveguide and L is the circumference of the microring. The input and output pulses are offset in time for ease of viewing.

Supplementary Figure 2

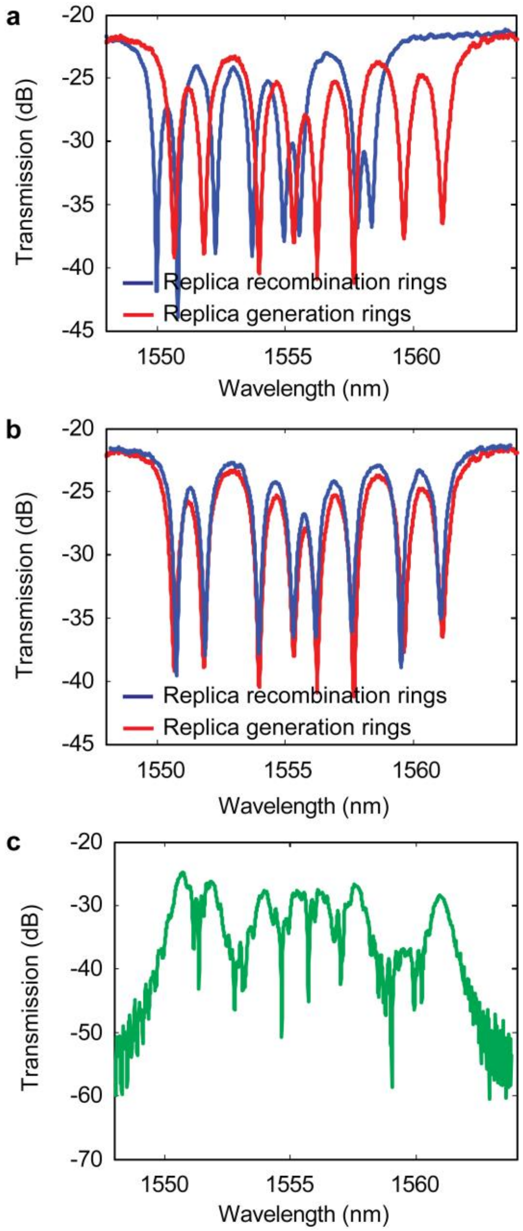

Supplementary Figure 2. Measured transmission spectra of the Si pulse shaper described in Fig. 1. The transmission spectra are taken at the common through and common drop ports of the replica generation rings and at the common through port of the replica recombination rings, respectively. (**a**) The through-port transmission spectra are taken before thermal resonance matching. (**b**) The through-port and (**c**) drop-port transmission spectra are taken after thermal resonance matching. The fibre-to-fibre loss of ~22 dB measured at the through ports is attributed to waveguide facet coupling loss.

Supplementary Figure 3

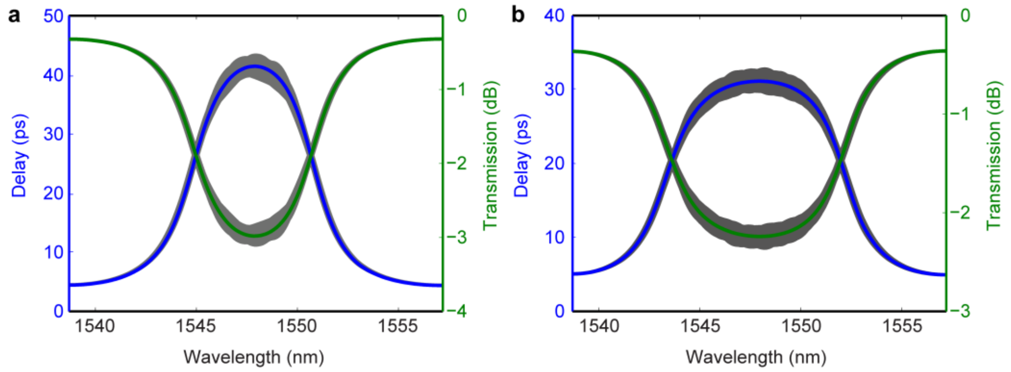

Supplementary Figure 3. Simulated delay and transmission spectra of two tuneable optical delay line designs using coupled-mode theory[2]. In both designs, all the microrings side couple to the bus waveguide at the same coupling gap of 100 nm (with a power coupling coefficient of $\kappa^2 \approx 0.6$) to satisfy the over-coupling condition. The propagation loss in the microrings is set as 10 dB/cm in the simulations. (**a**) The delay (blue) and transmission (green) spectra of an optical delay line that consists of 41 microrings with a radius increment step of 1 nm and a median radius of 5 µm. The simulated delay ranges from 4.5 ps to 41.5 ps, with the insertion loss increasing from 0.32 dB to 3 dB. To test the robustness of the design, we next assumed a random variation of ±2 nm in the radii of each of the 41 rings, which corresponds to the estimated precision of E-beam lithography in fabricating the delay line. 1000 simulations with random radius variations were performed and overlaid with that of the original design, showing a small variation of ~4 ps in delay and of 0.3 dB in transmission (shaded areas). (**b**) The delay and transmission spectra of another optical delay line design where the 41 microrings have a radius increment step of 1.5 nm and a median radius of 5µm. Both spectra are overlaid with the simulation results where random radius variations of ±2 nm in the radii of each of the 41 rings are incorporated.

Supplementary Figure 4

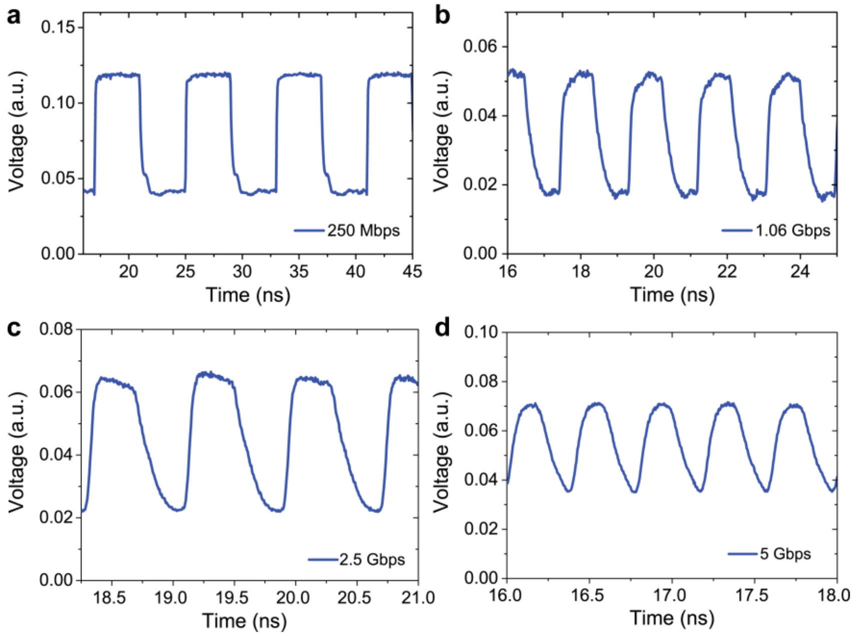

Supplementary Figure 4. Characterization of the silicon intensity modulator (embedded in the pulse shaper) with a continuous-wave (CW) laser input at 1549.7 nm. The modulator was tested at a modulation speed of (**a**) 250 Mbps, (**b**) 1.06 Gbps, (**c**) 2.5 Gbps, and (**d**) 5 Gbps, respectively. The modulator is the same design as those reported in Ref. [3] except that some modification is made to the traveling-wave electrode design to meet the testing requirement but at a price of a compromised modulation efficiency. In the experiment of rapidly reconfigurable AWG, we applied external electrical controls to the silicon chip using RF/DC probes, which required much more setup space than the wire bonding method. In the setup, we coupled light into and out of the chip through fibre butt coupling and the remaining setup space could only accommodate one 16-pin DC probe to reconfigure the pulse shaper and one RF probe to drive the modulator. However, driving a traveling-wave modulator typically requires using a pair of 'GSG' RF probes, one feeding the RF signal at one end of the electrodes and one terminating the RF wave at the other end. Thus using only one RF probe not only limits the number of modulators that can be incorporated into the shaper but also requires modification to the electrode design of the modulator. In our design, the electrodes were wrapped to one side of the modulator (See Fig. 4a in the main text) so that a single 'GSGSG' RF probe can feed and terminate the RF driving signal simultaneously. In addition, the thermal tuning element was also dropped in this modulator design due to lack of space for additional DC probing. As a consequence of the compromised electrode

design and lack of thermal tuning element, the extinction ratio of the modulator was limited to ~3:1 and the modulation speed was limited to 5 Gbps.

Supplementary Figure 5

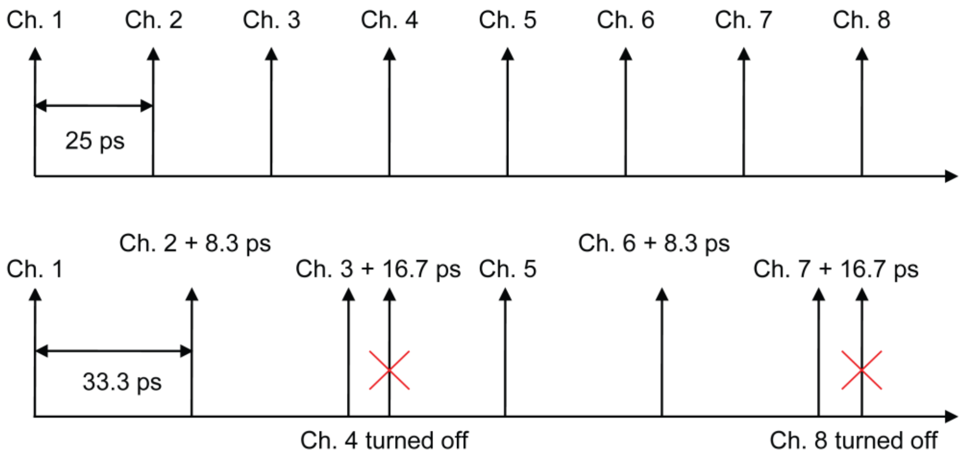

Supplementary Figure 5. Illustration of RF frequency synthesis by adjusting delays of some channels and disabling selected channels. The fixed delay is 25 ps between consecutive channels, corresponding to a 40 GHz fundamental RF tone. One can achieve a 33.3 ps channel spacing (30 GHz fundamental RF tone) by adjusting the 2nd and 3rd channel delays and disabling the 4th channel. Our architecture can easily disable any channel by shifting the recombination ring resonance away from that of the replica generation resonator. Experimental demonstrations are shown in Fig. 2e and Fig. 3b. Mathematically, since each channel can tune the delay (27 ps) more than the fixed channel spacing (25 ps), one can synthesize any RF waveforms as long as the highest frequency component is less than 1/(25 ps) = 40 GHz. To increase the highest frequency, one can reduce the fixed delay and increase the number of channels.

Supplementary Table 1

	Measurement (TE)	FDTD simulation (TE)	FDTD simulation (TM)
Free spectral range (nm)	11.8	12.6	14.2
Q factor	2600	2700	420
Power coupling coefficient κ^2	0.16	0.14	0.74

Supplementary Table 1. Measured and simulated characteristics of racetrack resonators in the foundry chip. The racetrack resonator has a bending portion of ~5 μm in radius and a straight portion of ~7.5 μm in length that side couples to the bus waveguide at a coupling gap of 200 nm. The experimental values of the free spectral range, *Q*-factor and power coupling coefficient κ^2 were retrieved from the transmission spectrum taken at the common through port of the replica generation rings, and they were averaged over 8 channels. The numerical values were determined using a 3D FDTD solver MEEP[1] for both the transverse-magnetic (TM) and transverse-electric (TE) modes of the racetrack resonators. By comparing the three groups of values, we confirmed that the racetrack resonators in the shaper worked in TE polarization as designed.

Supplementary References

1 Oskooi, A. F. *et al.* MEEP: A flexible free-software package for electromagnetic simulations by the FDTD method. *Comput. Phys. Commun.* **181**, 687-702 (2010).

2 Yariv, A. Universal relations for coupling of optical power between microresonators and dielectric waveguides. *Electron. Lett.* **36**, 321-322 (2000).

3 Wang, J., *et al.* Optimization and demonstration of a large-bandwidth carrier-depletion silicon optical modulator. *J. Lightwave Technol.* **31**,4119-4125 (2013).
